# How Much Is Enough? Minimal Responses of Water Quality and Stream Biota to Partial Retrofit Stormwater Management in a Suburban Neighborhood

**DOI:** 10.1371/journal.pone.0085011

**Published:** 2014-01-17

**Authors:** Allison H. Roy, Lee K. Rhea, Audrey L. Mayer, William D. Shuster, Jake J. Beaulieu, Matthew E. Hopton, Matthew A. Morrison, Ann St. Amand

**Affiliations:** 1 U. S. Geological Survey, Massachusetts Cooperative Fish and Wildlife Research Unit, University of Massachusetts, Amherst, Massachusetts, United States of America; 2 U. S. Environmental Protection Agency, Office of Research and Development, Cincinnati, Ohio, United States of America; 3 School of Forest Resources and Environmental Science and Department of Social Sciences, Michigan Technological University, Houghton, Michigan, United States of America; 4 SABIC, Mt. Vernon, Indiana, United States of America; 5 PhycoTech, Inc., St. Joseph, Michigan, United States of America; University of Sydney, Australia

## Abstract

Decentralized stormwater management approaches (e.g., biofiltration swales, pervious pavement, green roofs, rain gardens) that capture, detain, infiltrate, and filter runoff are now commonly used to minimize the impacts of stormwater runoff from impervious surfaces on aquatic ecosystems. However, there is little research on the effectiveness of retrofit, parcel-scale stormwater management practices for improving downstream aquatic ecosystem health. A reverse auction was used to encourage homeowners to mitigate stormwater on their property within the suburban, 1.8 km^2^ Shepherd Creek catchment in Cincinnati, Ohio (USA). In 2007–2008, 165 rain barrels and 81 rain gardens were installed on 30% of the properties in four experimental (treatment) subcatchments, and two additional subcatchments were maintained as controls. At the base of the subcatchments, we sampled monthly baseflow water quality, and seasonal (5×/year) physical habitat, periphyton assemblages, and macroinvertebrate assemblages in the streams for the three years before and after treatment implementation. Given the minor reductions in directly connected impervious area from the rain barrel installations (11.6% to 10.4% in the most impaired subcatchment) and high total impervious levels (13.1% to 19.9% in experimental subcatchments), we expected minor or no responses of water quality and biota to stormwater management. There were trends of increased conductivity, iron, and sulfate for control sites, but no such contemporaneous trends for experimental sites. The minor effects of treatment on streamflow volume and water quality did not translate into changes in biotic health, and the few periphyton and macroinvertebrate responses could be explained by factors not associated with the treatment (e.g., vegetation clearing, drought conditions). Improvement of overall stream health is unlikely without additional treatment of major impervious surfaces (including roads, apartment buildings, and parking lots). Further research is needed to define the minimum effect threshold and restoration trajectories for retrofitting catchments to improve the health of stream ecosystems.

## Introduction

Rapid urbanization and the ongoing conversion of landscapes from natural habitats to industrial, commercial, and residential land uses to support a growing human population remain the most salient threats to natural ecosystems [1]–[3]. Aquatic ecosystems that drain urban areas are particularly vulnerable due to their low position in the landscape [4]. In most urban and suburban areas, untreated stormwater runoff from impervious surfaces is typically routed directly into rivers, lakes, and oceans. This conventional design of urban drainage systems reflects concerns about human health and safety, but largely ignores threats to aquatic ecosystem health that stem from stormwater runoff [5], [6].

The urban stream syndrome describes changes in stream ecosystems associated with urbanization, a subject that has been increasingly studied in the last few decades (see reviews by [7]–[9]). These changes primarily arise from stormwater runoff from impervious cover—particularly impervious cover that is directly connected to streams by stormwater pipes [6]— which alters stream hydrology, water chemistry, and biotic communities. High magnitude, flashy flows in urban streams can scour stream beds and erode stream banks, thus reducing habitat quality. Furthermore, the extreme high flows can wash out aquatic biota and low lying riparian vegetation, whereas reduced base flows can reduce in-stream habitat and alter stream ecosystem function [10]. Runoff that enters urban and suburban streams often contains increased toxicants, ions, and nutrients, along with higher temperatures, reduced oxygen saturation, and organic material that can alter biotic structure and ecosystem function (production, nutrient uptake, leaf breakdown, etc.) relative to streams in natural landscapes [7]. While there are other catchment stressors (e.g., point sources, septic systems, riparian degradation) and in-stream stressors (e.g., impoundments, water withdrawals, stream burial) associated with urbanization [9], stormwater runoff is a dominant source of impairment of ecological structure and function in most urban catchments. As such, comprehensively managing stormwater runoff with the goals of mimicking pre-disturbance flow regimes, improving water quality, and ultimately improving ecosystem health is a leading approach to urban stream restoration [5], [6], [11], [12].

There is an increasing movement throughout the world to address runoff through decentralized stormwater management [6], [13]–[15]. This management approach can include small-scale tools that capture and detain (e.g., detention and retention basins), infiltrate (e.g., pervious pavements, rain gardens), and filter (e.g., biofiltration swales, wetlands) runoff on individual parcels throughout a landscape [16]–[18]. These tools are implemented through new development (often referred to as low impact development, LID) [15] or retrofitting of already-developed areas.

To date, there have been no reports of the effectiveness of decentralized stormwater management for improving stream water quality and biota in suburban catchments. Most assessments are limited to measurements of hydrology and water quality within individual treatment practices [18], [19] (see also www.bmpdatabase.org) and modelled effects of installations throughout catchments [20]–[22]. There have been some catchment-scale studies comparing LID and conventional practices in new developments [23], [24], but no catchment-scale retrofits of existing developments. This is due in part to the distribution of impervious surfaces within catchments, and the legal, economic, and logistical difficulty of implementing stormwater management practices at a scale appropriate for improvement [25], [26]. The research presented here and another study currently underway in Australia [27] are, to our knowledge, the first attempts at assessing stream responses to retrofit stormwater management at the catchment scale.

Like many large, older US cities, the metropolitan area of Cincinnati, OH has an aging stormwater infrastructure that uses common combined sewer overflows, which lead to both an environmental and legal (e.g., consent decree) need to address stormwater management [28]. Thurston et al. [29] used Cincinnati to demonstrate that decentralized stormwater abatement was likely to be less expensive than a centralized (e.g., deep tunnel) solution to the water quality and quantity problem. Thus, a multidisciplinary study was designed that 1) assessed the legal, economic, and scientific challenges associated with decentralized stormwater management [25], and 2) developed and tested a stormwater management strategy within a small, suburban catchment. Rain barrels (up to four per property) and rain gardens (one per property) were offered to eligible residents (i.e., owner-occupied and within the experimental area) through a voluntary, reverse auction where bids consisted of the stormwater management practice(s) and a financial subsidy (if desired). The lowest cost bids at locations with the highest potential environmental benefits were prioritized. Winning homeowners received the bid amount, free stormwater management practices, installation, and maintenance for three years [30]. The voluntary nature of the auction avoided private property rights issues while providing financial incentives to property owners for installation and increasing stakeholder ownership [25]. The project placed 81 rain gardens and 165 rain barrels on ca. 30% of the eligible properties within the headwaters of the Shepherd Creek catchment in 2007 and 2008 [30]. The rain barrels resulted in an overall decrease in directly connected impervious area (DCIA) from 7.4% to 7.0% in the catchment, and 11.6% to 10.4% (Sub1), 9.0% to 8.1% (Sub2), and 7.3% to 7.1% (Sub3) in the Experimental subcatchments (Table 1). Rain gardens did not change the DCIA, but offered additional capacity to capture overland runoff.

**Table 1:** Catchment area and land cover based on cumulative piped catchments draining to each site.

Site	Total Area (ha)	Forest Cover^1^ (%)	TIA^2^ (%)	DCIA^3^ (%)			2007 Installations^4^			2007 & 2008 Installations		
				Before (<2007)	During (2007–08)	After (2008)	Barrels	Gardens	Density (#/ha)	Barrels	Gardens	Density (#/ha)
Sub1	28.0	43.8	19.9	11.6	11.2	10.4	50	20	2.5	84	33	4.2
Sub2	57.9	46.3	16.3	9.0	8.7	8.1	68	27	1.6	123	55	3.1
Sub3	68.9	68.0	13.1	7.3	7.2	7.1	32	23	0.80	42	26	1.0
Catch	183	62.6	13.1	7.4	7.2	7.0	100	50	0.82	165	81	1.3
Sub4	24.9	66.4	11.2	5.4	5.4	5.4	1	1	0.08	1	1	0.08
Sub5	34.8	64.2	12.1	7.3	7.3	7.3	0	0	0.00	0	0	0.00

^1^ Forest cover is based on topographic catchment.

^2^ Total impervious area (%) includes all impervious surfaces identified by air photos and site visits [38].

^3^ Directly connected impervious area (DCIA) was calculated by subtracting the rooftop area draining to rain barrels installed in 2007 and 2008.

^4^ Stormwater management installations includes number of rain barrels and rain gardens installed in 2007 and overall (2007 & 2008), and the total density of barrels and gardens within each subcatchment.

The objective of our study was to determine if the retrofit stormwater management imposed as a result of the economic auction would result in measurable shifts in the ecological condition of streams in the Shepherd Creek catchment. We assessed habitat conditions, baseflow water quality, and the biological assemblage (periphyton and macroinvertebrates) at various sites along tributary streams within the catchment. For the hydrometric portion of the Shepherd Creek study, Shuster & Rhea [31] demonstrated a small but significant effect of decentralized stormwater management on stream hydrology, reflected by a decrease in runoff volume in treatment subcatchments. Given the small change in hydrology and small reduction in % DCIA, combined with levels of impervious cover above published thresholds of impairment [6], [32], we expected minimal or no responses of water quality and biota to stormwater management.

## Methods

### Study design

**Figure 1:**
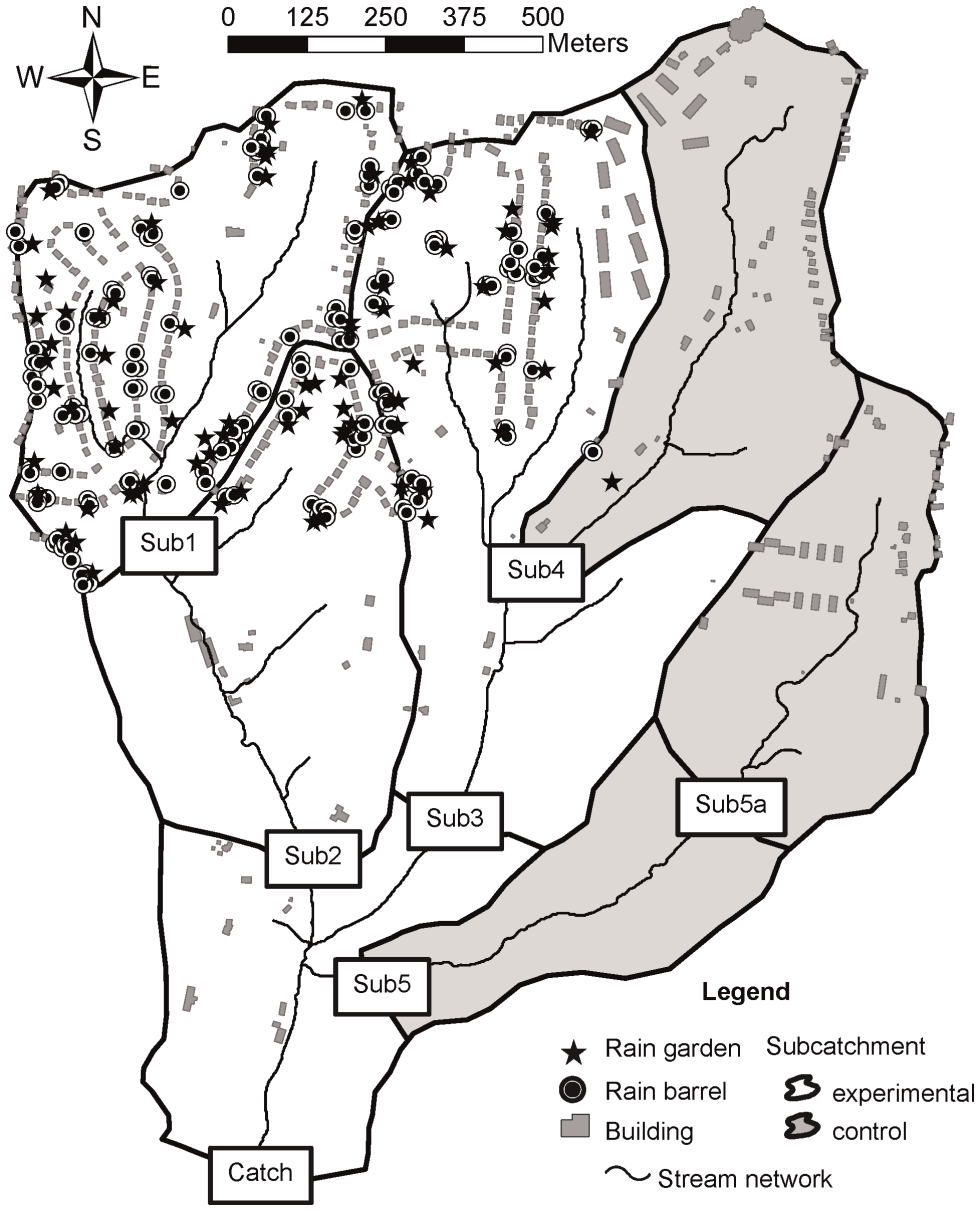
Shepherd Creek catchment and subcatchments in Cincinnati, Ohio (USA). Sub1 was nested within Sub2, Sub4 was nested within Sub3, and all of the subcatchments drained to the catchment outlet (Catch). Sub 5a was discontinued in 2006. Rain barrels and rain gardens were installed throughout the catchment except in Sub4 and Sub5.

We used a modified before–after–control–intervention (BACI) study design, where the intervention was the installation of treatments (rain barrels and rain gardens) on select parcels within the catchment. The modified paired-catchment BACI design relied on comparison of the difference between responses of Control and Experimental subcatchments for three periods to determine if significant treatment effects were present [33]–[35]. Typically only a pre-treatment and experimental period are used in a BACI design, but because implementation of the treatments spanned 16 months, we also included a transition period. Six study sites were sampled within the Shepherd Creek catchment, including four Experimental sites (Sub1, Sub2, Sub3, Catch) and two Control sites (Sub4, Sub5; Figure 1). Access to field sites Sub1, Sub2, Sub3, and Catch was granted by private landowners. Sites Sub5 and Sub5a were in the Mt. Airy Forest, and permission was granted by the Cincinnati Park Board. Sub4 was in the road right-of way. Multiple Experimental and Control sites were used to minimize potential confounding of location-specific differences with treatment effects [36], [37]. Total impervious area (TIA) in the Shepherd Creek catchment was 13.1%, with just over half (7.4%) of the TIA directly connected to stormwater or combined sewer pipes (Table 1) [38]. The five subcatchments (24.9–68.9 ha) ranged from 11.2–19.9% TIA, with a corresponding range of 43.8–68.0% forest cover (Table 1).

Sites were sampled five times per year from April through October from 2003 through 2010, with a few exceptions. Sub5 was moved downstream in 2005 due to lack of sufficient flow at Sub5a. Sub4 was added in April 2005 to provide an additional Control, and Sub1 was discontinued in October 2009 following sewer repair that drastically changed stream baseflow hydrology. Stormwater treatment installation occurred in June through September 2007 (Phase 1) and July through September 2008 (Phase 2). Sample periods were therefore designated as Before (prior to June 2007), During (June 2007 through September 2008), and After (October 2008 and later), in respect to treatment installation. There was no new road or building construction in the study area during the study period, but there were some potentially significant events, including: road and sewer repair in Sub1 in 2006 and 2008, roadside vegetation clearing along Sub4 in summer 2007, and invasive riparian plant removal in Sub5 in summer 2007.

### Physical and chemical characteristics

Basic morphometric, geomorphic, and water quality parameters were measured five times/year within the 61-m sample reach during biotic sampling. We calculated approximate values for average width, average depth, wetted area, and surface velocity based on field measurements. Additional physical attributes included estimates of: % riffle, % pool, % run, large wood density, % small wood, % large wood, % detritus, bed texture (% bedrock, cobble, gravel, sand, silt), and % canopy cover following the EPA Rapid Bioassessment Protocols (RBP) Physical Characterization data sheet [39]. Water quality measurements were taken with a YSI 6600 data sonde (YSI, Inc., Yellow Springs, OH, USA), and included: water temperature, conductivity, dissolved oxygen, pH, oxidation-reduction potential, and turbidity. YSI data sondes were calibrated within 24 hours of use and tested immediately following return from the field. Measurements from water quality probes that did not pass post-deployment calibration checks were not used. Finally, we calculated two visual assessment habitat evaluation scores: EPA's Rapid Bioassessment Protocols Quantitative Habitat Assessment (QHEI) [39], and the Primary Headwater Habitat Evaluation Form (HHEI) [40] that is specifically designed for streams with water depths <40 cm.

Water quality sampling was also conducted monthly during baseflow conditions. In addition to measuring the water quality parameters described above, grab water samples were filtered with a 0.45-µm filter for total dissolved phosphorus (TDP), dissolved organic carbon (DOC), and dissolved metals (Al, Fe, Mn, Cu, Zn). TDP and DOC were preserved with sulfuric acid, and dissolved metals were preserved in nitric acid. Unfiltered grab samples were collected for analysis of nutrients (nitrate/nitrite nitrogen, ammonium nitrogen, dissolved inorganic nitrogen, total Kjeldahl nitrogen, total phosphorus, ortho-phosphate, TDP), total organic carbon (TOC), total recoverable metals (Al, Fe, Mn, Cu, and Zn), and base cations (Na, Mg, K, Ca). Nutrients and TOC were preserved with sulfuric acid, and metals and base cations were preserved with nitric acid. Suspended sediment concentration (SSC), anions (Cl, Br, F, SO_4_, NO_3_, and ortho-PO_4_), and alkalinity samples were unpreserved. For SSC, the 250-mL sample was filtered through a pre-washed and pre-weighed 1.5-µm glass-fiber filter, dried to a constant weight, and re-weighed following ASTM Method D3977-97 [41]. Analyses for nutrients, metals, anions, and cations were performed by EPA Region 5 Central Regional Laboratory (Chicago, IL) using standard EPA protocols as follows: EPA Method 353.2 for nitrate/nitrite nitrogen, EPA Method 350.1 for ammonia nitrogen, EPA Method 351.2 for total Kjeldahl nitrogen, EPA Method 365.4 for total and dissolved phosphorus, EPA Method 415.1 for total organic carbon, EPA Methods 200.2 (digestion) and 200.7 (analysis) for metals and base cations, and CRL Method AIG045 for anions, based closely on EPA Method 300.1.

### Periphyton

Periphyton samples were collected from submerged rocks throughout the 61-m study reach. Cobbles were removed from the stream and a 13.2-cm^2^ area on each rock, designated with a PVC ring, was brushed with a toothbrush for 2 min. Rocks and brushes were then rinsed with stream water into a 500-mL bottle. Algae from all rocks within a reach were composited into a single bottle and placed in the dark on ice.

In the laboratory, 20–30 mL of the periphyton slurry was filtered onto each of two glass fiber filters and frozen for subsequent analysis of chlorophyll *a* using a multi-wavelength spectrophotometer following EPA's Method 446.0 [42]. An additional 50 mL of sample was preserved in 1% gluteraldehyde for biomass analysis. The sample was later filtered onto a pre-ashed glass fiber filter (47 mm, PALL Type A/E, 1-µm pore size). Filters were dried at 105°C to a constant weight, weighed for dry weight, ashed in a muffle furnace for 1.5 hours at 500°C, wetted, re-dried at 105°C, and re-weighed to obtain ash-free dry mass (AFDM). The remaining algal sample was preserved in 1% gluteraldehyde for identification. All algae (diatoms and soft algae) were identified and enumerated to the genus level by PhycoTech, Inc., consistent with Standard Methods 10200 and 10300 [43]. Three permanent slide mounts were made with 2-hydroxypropyl methacrylate (HPMA), and all slides were examined using a stratified counting procedure (200× and 400× for soft algae, 1000× for diatoms and picoalgae) to a minimum of 400 natural units [44].

Algal indices were calculated based on densities of cells per cm^2^ and included total density, density of major orders (Bacillariophyta, Chlorophyta, and Cyanophyta), relative proportions of the major orders, taxa richness, Shannon diversity, and percent of sample in the dominant taxon. A periphyton index of biotic integrity (PIBI) developed for the mid-Atlantic region of the United States was calculated that incorporated nine metrics (phosphatase activity metric excluded) [45].

### Macroinvertebrates

Macroinvertebrates were collected using two methods: 1) a triangular dip net (500-µm mesh) used to collect a composited, multi-habitat sample in the entire 61-m reach, and 2) a bucket sampler (0.053-m^2^) within three replicate depositional/riffle habitats. The net samples were considered ideal for capturing macroinvertebrate diversity and relative abundance [39], whereas the bucket samples were used to determine macroinvertebrate densities in riffle habitats that are most sensitive to disturbance [46]. Net samples were collected five times/year in conjunction with periphyton and habitat sampling, and bucket samples were collected during three sampling events per year (spring, summer, autumn). Bucket samples were taken by pushing an open bucket into the bed sediment, surrounding the bucket-sediment interface with a custom-made canvas skirt to enclose the area, and scrubbing each large rock into the water contained in the bucket. The bed sediment was then disturbed for 10 sec. using a trowel, followed by 10 sec. of sweeping with a small dip net (500-µm mesh), and repeated for a total of three times. All samples were emptied into wash basin, elutriated, poured through a 500-µm sieve, and preserved in 70% ethanol.

In the lab, macroinvertebrates were subsampled to a minimum of 300 individuals and identified to lowest possible taxonomic unit (typically genus or species), enumerated, and measured (body length or shell width). All midges (Diptera: Chironomidae) and oligochaetes were slide-mounted for identification. To address differences in taxonomic resolution among three contractors, we lumped taxa to a common taxonomic level (e.g., genus or family) or assigned lower classification levels as appropriate (e.g., where there was only one genus in a family found at our sites). Macroinvertebrate biomass was calculated using published length-mass relationships (e.g., [47]) to generate AFDM for each taxon. Several macroinvertebrate indices were calculated for analysis. Abundance, relative abundance, richness, and biomass were calculated for Chironomidae, EPT (Ephemeroptera, Plecoptera, and Trichoptera), insects only, and all taxa. We calculated the abundance, relative abundance, and biomass of the isopod Asellidae (typically the most abundant taxon in each sample), the proportional abundance and biomass of the dominant taxon in each sample, and Shannon diversity.

### Statistical analysis

Data analyses were performed using SAS version 9.2 (SAS Institute, Cary, NC, USA). Prior to any analysis, each analyte (e.g., water quality parameters, habitat measures, periphyton and macroinvertebrate indices) was screened for outliers using scatter plots and histograms; Box-Cox transformed for normality using SAS proc Transreg; normalized using SAS proc Standard; and analyzed using SAS proc Mixed and SAS proc HPmixed. Less than 10 percent of data were excluded as outliers at this stage. Each analyte was then evaluated separately using a simplified “screening” model including study period (Period as Before, During, or After treatment implementation), sample site (Site), and sample group (Group as Control or Experimental) with the “influence” option selected to identify suspicious data points. For the most part, we could not differentiate outliers due to human error from system noise. We believe that the outliers do more to obscure real signals than reflect actual conditions, and therefore have we have omitted them from further analysis.

After data cleaning was completed, separate analyses were performed for each analyte to assess the responses of individual variables to treatment using SAS proc Mixed. Model parameters included Period, Site, Group, Group*Period interaction, and sampling round (Round as sampling dates grouped in 7-day windows) (Table 2). Period and Group were coded in the model as a numerical, fixed main effects, Site was coded as a fixed effect nested within Group, and Round was coded as a random effect. The Group*Period interaction was used to assess for significance of treatment effects.

**Table 2:** Variables used in statistical models.

Variable	Type	Description
Period	fixed, numeric	study period (Before treatment = 0, During treatment = 0.5, After treatment = 1)
Site	fixed, class	study sites (Sub1-5, Catch); nested in Group
Group	fixed, numeric	Control (Sub4, Sub5 = 0) and Experimental (Sub1, Sub2, Sub3, Catch = 1) sites
Round	random, class	sample collection dates grouped in 7-day windows
Sample	random, class	sample event (year and month) (ordination analyses only)
Group*Period	fixed, class	interaction of study period and group; indicates significant effect of stormwater treatment
Axis	fixed, class	NMS axis number (ordination combined axes analysis)
Axis*Period	fixed, class	interaction of NMS axis and study period (ordination combined axes analysis)
Axis*Group	fixed, class	interaction of NMS axis and study sites (ordination combined axes analysis)

We used non-metric multidimensional scaling (NMS) to ordinate taxonomic data and express differences in assemblage structure across samples. Species-specific periphyton abundances and macroinvertebrate abundances and biomass (for bucket samples only) were log (x+1) transformed and extreme biomass outliers (3 samples) were removed prior to ordination. The NMS ordination was configured with the Sorensen distance measure and step down in dimensionality, and run in PC-ORD™ (Version 6, MjM Software Design, Gleneden Beach, OR, USA). The ordination axes were tested for fixed effects of Period, Group, Site (nested in Group), and Group*Period interactions, and random effect of Sample (month, year) using SAS proc Mixed. The combined axes for each analyte type (periphyton, macroinvertebrate abundance, and macroinvertebrate biomass) ANOVA included fixed effects for Period, Group, Group*Period, Axis, Axis*Period, and Axis*Group. Due to the ordination step, the statistical model was slightly altered, but equivalent to that used for non-ordinated data. In all cases, a significant Group*Period interaction indicated a significant effect of stormwater treatment installation (Table 2).

For all results, we used a *P*<0.05 cut-off for designating significance. Following the recommendations of Moran [48] for diverse ecological data and in the interest of maintaining detailed analyses, we did not correct for multiple comparisons (e.g., using sequential Bonferroni). Therefore, we caution the reader to tend toward a more conservative interpretation of tests that may be affected by Type I error, even for *P*-values<0.05. All raw data and SAS codes are available in EPA's STORET database (http://www.epa.gov/storet/).

## Results

### Landscape conditions, habitat, and water quality

Most of the headwater streams had a mix of gravel, cobble, and boulder substrate with high amounts of fine sediment deposition in the pools. The QHEI habitat scores reflected the mix of substrates and high proportion of riffle habitats, although most sites had suboptimal conditions due to high sediment deposition, poor vegetative protection, and moderately unstable banks (Table 3). Baseflow water quality varied considerably across sites and seasons, but on average streams had elevated nutrients (nitrate = 1.40 mg/L; total dissolved phosphorus = 0.620 mg/L), and high conductivity. Both natural (e.g., limey parent material as calcium = 99.4 mg/L) and anthropogenic (e.g., road salting and domestic wastewater as chloride = 147 mg/L) sources of ions likely contributed to high conductivity (Table 3).

**Table 3:** Physical and chemical variable summary statistics and ANOVA results for Group*Period interaction.

Variable	Lambda^1^	N	Min	Max	Mean	St Dev	*P* 2	
Habitat^3^								
DO (mg/L)	1.45	206	0.350	13.15	7.11	2.98	0.087	
HHEI score^4^	1.80	204	51.0	99.0	81.3	8.55	0.722	
Oxidation reduction potential (mV)	1.35	190	1.80	392	195	77.3	0.501	
Conductivity (mS/cm)	−1.55	210	0.280	2.53	1.00	0.394	<0.001	***
Temperature (°C)	1.90	210	5.68	25.3	15.8	4.68	0.218	
Turbidity (NTU)	−0.250	154	0.100	19.9	8.71	5.40	0.042	*
Closed canopy (%)	2.50	199	5.00	90.0	60.9	22.4	<0.001	***
Mean depth (m)	−6.25	201	0.019	0.600	0.074	0.066	0.067	
Filamentous algae (score)	−1.25	88	1.00	4.00	1.76	0.830	0.017	*
Wetted reach area (m^2^)	−1.30	203	4.30	366	100.07	61.75	0.744	
Riffle habitat (%)	1.90	179	5.00	90.0	50.9	20.2	0.331	
QHEI score^5^	1.50	204	55	175	113.97	23.53	0.702	
Water Quality^6^								
Alkalinity (mg CaCO_3_/L)	−7.00	293	100	350	251	40.7	0.240	
Chloride (mg/L)	−1.80	273	0.100	856	147	125	0.228	
Calcium (mg/L)	3.10	279	1.40	182	99.4	42.4	0.010	*
Dissolved organic carbon (mg/L)	0.55	282	1.90	35.2	9.95	7.59	0.216	
Iron (mg/L)	−5.70	285	0.002	3.42	0.287	0.342	<0.001	***
Magnesium (mg/L)	−1.00	255	0.100	35.4	19.4	6.22	0.104	
Nitrate (mg/L)	−6.55	271	0.020	10.7	1.40	1.58	0.858	
pH	0.500	278	6.07	10.2	7.79	0.507	0.623	
Sulfate (mg/L)	−5.20	242	0.100	222	83.3	37.6	0.015	*
Suspended sediment concentration (mg/L)	−1.75	286	−8.22	45.9	7.84	8.75	0.842	
Total dissolved phosphorus (mg/L)	−2.20	277	0.040	0.620	0.185	0.099	0.539	
Total organic carbon (mg/L)	−2.05	281	1.80	37.3	11.6	8.74	0.526	
Temperature (°C)	2.40	272	0.090	25.2	12.9	7.02	0.831	
Zinc (dissolved) (µg/L)	−2.10	232	0.010	127	21.0	18.4	0.482	

^1^ Lambda is value for the exponential transformation.

^2^***P<0.001, *P<0.05.

3 Habitat variables (including some water quality variables) were sampled five times per year during biotic sampling events.

4 HHEI score from Ohio Environmental Protection Agency Primary Headwater Habitat Evaluation Index [40].

5 QHEI score from Rapid Bioassessment Protocols Quantitative Habitat Assessment for high gradient streams, and filamentous algae score (range 0–4) is from RBP benthic macroinvertebrate field sheet [39].

6 Water quality variables were sampled monthly during baseflow conditions.

**Figure 2:**
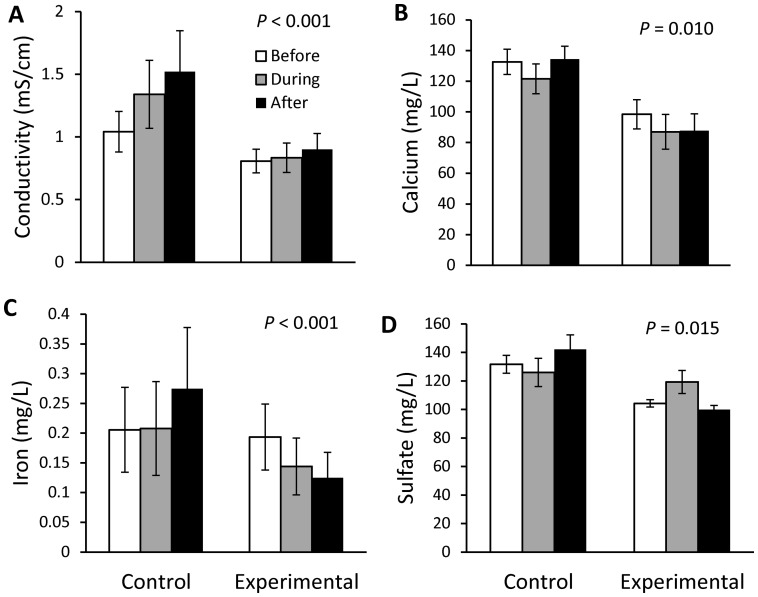
Water chemistry before, during, and after treatment for control and experimental sites. Mean (± SE) back-transformed values are reported. Conductivity (A) was sampled during seasonal biotic monitoring, and calcium (B), iron (C), and sulfate (D) were sampled during monthly baseflow water quality monitoring. *P*-values reflect results of ANOVA for Group*Period interaction for the strongest models (Table 3).

**Figure 3:**
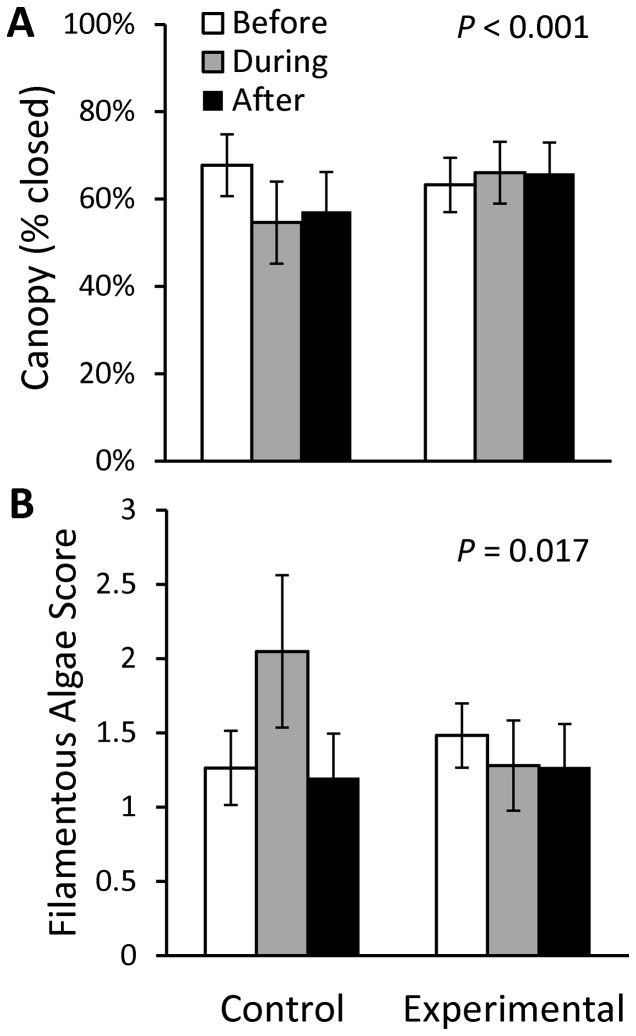
Canopy cover (A) and filamentous algae score (B) before, during, and after treatment for control and experimental sites. Mean (± SE) back-transformed values are reported based on qualitative data collected during biotic sampling. *P* values reflect results of ANOVA for Group*Period interaction (Table 3).

There was a significant effect of treatment (rain garden and rain barrel installations) on several habitat and water quality parameters (Table 3). Conductivity (Fig. 2A), iron (Fig. 2C), and sulfate (Fig. 2D) increased in the Control sites through time, with no apparent change or a decrease (iron) over the corresponding periods in Experimental sites. Calcium concentrations were similar in Control sites Before and After installations, but decreased in Experimental sites through time (Fig. 2B). Canopy cover was lower in the During and After periods (vs. Before), corresponding to the vegetation removal that occurred at the Control sites in 2007 (Fig. 3A). The qualitative filamentous algae score was much higher in the During period than Before and After treatment at the Control sites, whereas the Experimental sites did not experience a similar fluctuation through time (Fig. 3B).

### Periphyton

Over seven years, 107 periphyton genera were identified. Average periphyton densities were highest in April prior to leaf-out (15.4 M cells cm^−2^) and lowest May/June (8.0 M cells cm^−2^). Chlorophyll *a* was also highest in April (52.6 mg m^−2^) compared to other seasons (average range: 5.31–9.29 mg m^−2^). In contrast, algal AFDM was similar across seasons (range 43.2–49.2 g m^−2^), but varied across sites (range 38.3–62.2 g m^−2^, Sub3–Sub5). The periphyton were numerically dominated by Cyanophyta (cyanobacteria) in the family Chroococcaceae, encompassing 83% of the total cell density. This dominance was consistent across sites (site averages 78–85%), but increased throughout the growing season from 63% in April (before leaf-out) to 92–94% in June through October. Numerically, the cyanobacteria were dominated by small pico-periphyton <2 µm. Within Bacillariophyta (diatoms), *Navicula* (51.6%), *Nitzchia* (14.0%), *Amphora* (9.3%), *Gomphonema* (9.1%), and *Achnathes* (5.7%) were the most common genera. Average Shannon diversity (1.43–1.54) and PIBI scores (21.4–24.8) were similar across sites, and generally reflected degraded stream conditions.

**Figure 4:**
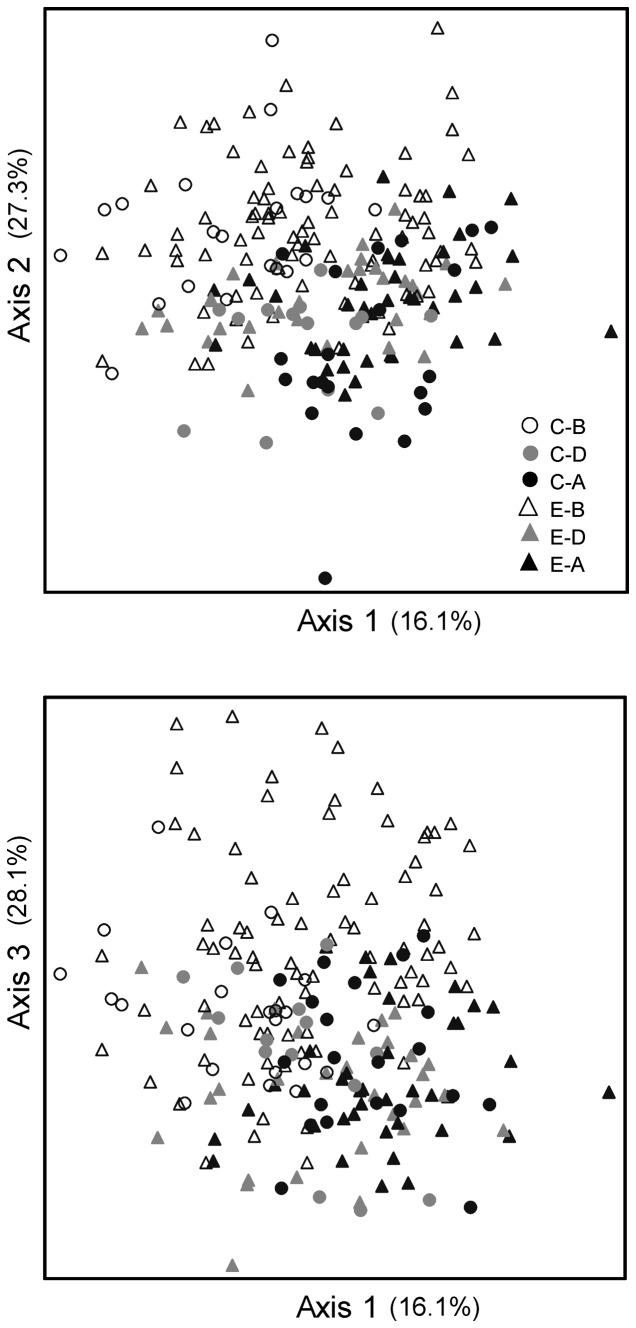
Non-metric multidimensional scaling ordination for periphyton abundances. Assemblages were different for Control (C) and Experimental (E) sites (Axis 2) and comparing Before (B), During (D), and After (A) installation of stormwater management (Axes 1, 2, and 3; Table 5). The 3D solution explained 71.5% of the variation, and the final stress was 19.3.

Analysis of the five individual periphyton metrics that met the statistical criteria revealed no significant treatment effects (Table 4). NMS ordination of cell densities of periphyton taxa revealed a visible shift between samples Before, During, and After treatment installations in the 3-dimensional solution (Fig. 4). Further analysis of the individual axes showed significant Period effects for all three axes separately, and a significant effect of Group (Control vs. Experimental) for axis 2 (Table 5). However, there were no significant effects of Group*Period (treatment) for any of the NMS axes separately or combined (Table 5).

**Table 4:** Biotic variable summary statistics and ANOVA results for Group*Period interaction.

Variable	Lambda^1^	N	Min	Max	Mean	St Dev	*P* 2	
Periphyton								
% Dominant diatom	−0.400	218	20.2	100	55.0	17.9	0.375	
% Motile diatom	1.00	212	3.90	100	52.2	25.8	0.602	
PIBI^3^	1.75	216	0.100	37.8	21.3	6.79	0.415	
Shannon diversity	0.050	218	0.740	2.78	1.48	0.376	0.218	
% Eutraphentic diatom	3.00	210	4.30	100	63.2	26.0	0.402	
Macroinvertebrates^4^								
*Abundance (bucket)*								
% Chironomidae	−2.75	102	1.04	89.9	29.6	24.8	0.088	
Chironomidae richness	0.550	102	1.00	19.0	8.87	4.38	0.053	
% EPT^5^	−7.00	77	0.101	35.4	5.17	6.69	0.324	
EPT richness	−3.70	77	0.500	8.00	2.79	2.13	0.786	
Shannon diversity	2.35	104	0.220	3.08	2.14	0.561	0.267	
Insect richness	1.20	104	3.00	33.0	16.1	6.22	0.002	**
% Asellidae	−3.15	103	0.179	83.9	26.8	24.5	0.400	
Total richness	0.800	104	7.00	45.0	24.0	6.70	0.009	**
*Abundance (net)*								
% Chironomidae	−4.20	215	0.276	96.2	21.1	21.1	0.254	
Chironomidae richness	−1.05	215	1.00	23.0	7.18	4.16	0.705	
EPT richness	−2.25	144	0.500	7.00	2.42	1.51	0.420	
Shannon diversity	1.70	220	0.176	3.14	1.72	0.702	0.143	
Insect richness	−0.45	220	1.00	32.0	13.9	6.56	0.932	
% Asellidae	0.050	210	0.293	97.4	44.5	32.3	0.503	
Total richness	−0.15	220	5.00	42.0	21.3	7.53	0.924	
*Biomass (bucket)*								
Chironomidae richness	0.700	102	1.00	20.0	9.21	4.52	0.058	
% EPT	−7.00	76	0.007	70.4	7.72	12.1	0.478	
Shannon diversity	1.05	104	0.188	2.63	1.46	0.512	0.019	*
% Insecta	−1.15	104	0.561	96.5	41.0	30.9	0.318	
% Dominant taxon	−0.550	104	19.4	97.3	55.0	19.3	0.029	*
% Asellidae	−2.70	103	0.015	85.4	28.4	26.3	0.243	

1 Lambda is value for the exponential transformation.

2 **P<0.01, *P<0.05.

3 PIBI is the Periphyton Index of Biotic Integrity [45].

4 Macroinvertebrate variables were calculated separately for multi-habitat net samples (based on abundance data) and bucket samples in riffle habitats (represented as abundance and biomass).

5 EPT represents taxa in the orders Ephemeroptera, Plecoptera, and Trichoptera (considered sensitive to disturbance).

**Table 5:** ANOVA results for ordination axes.

Effect^1^	Axis	*P* 2		Effect	Axis	*P*	
Periphyton							
Group	1	0.151		Site(Group)	1	0.495	
Group	2	0.006	**	Site(Group)	2	0.232	
Group	3	0.510		Site(Group)	3	0.108	
Group	All	0.370		Site(Group)	All	–	
Period	1	0.014	*	Group*Period	1	0.913	
Period	2	<0.001	***	Group*Period	2	0.146	
Period	3	<0.001	***	Group*Period	3	0.311	
Period	All	0.755		Group*Period	All	0.983	
Macroinvertebrate Abundance^3^							
Group	1	0.085		Site(Group)	1	0.434	
Group	2	<0.001	***	Site(Group)	2	0.009	**
Group	3	<0.001	***	Site(Group)	3	<0.001	***
Group	All	0.032	*	Site(Group)	All	–	
Period	1	0.295		Group*Period	1	0.497	
Period	2	0.532		Group*Period	2	0.977	
Period	3	0.163		Group*Period	3	0.002	**
Period	All	0.024	*	Group*Period	All	0.815	
Macroinvertebrate Biomass							
Group	1	0.291		Site(Group)	1	0.006	**
Group	2	<0.001	***	Site(Group)	2	<0.001	***
Group	3	0.463		Site(Group)	3	0.311	
Group	All	0.237		Site(Group)	All	–	
Period	1	0.008	**	Group*Period	1	0.624	
Period	2	0.231		Group*Period	2	0.097	
Period	3	0.369		Group*Period	3	0.934	
Period	All	<0.001	***	Group*Period	All	0.989	

1 See Table 2 for variable descriptions.

2 ***P<0.001, **P<0.01, * *P*<0.05. – indicates effect not tested in combined axis model.

3 Macroinvertebrate abundance and biomass were based on bucket samples in riffle habitats.

### Macroinvertebrates

We collected 189 unique macroinvertebrate taxa across all samples and sites. Assemblages were dominated by the Asselid isopod *Lirceus* that composed 60% of the abundance of all net samples and 39% of bucket samples. Oligocheata worms (8.7% and 28.8%, respectively) and the chironomid Tanytarsus (5.1% and 12.6%, respectively were the second and third most abundant taxa, respectively). Other common taxa (composing >2% of the abundance) included the chironomids *Diamesa*, *Paratendipes*, and *Orthocladius*, and Ostracoda crustaceans. On average (± SD), the bucket samples were composed of 29.6±24.8% Chironomidae, 26.8±24.5% Asellidae, and 5.2±6.7% EPT taxa (Table 4). Across the six sites, there was a range in average total richness (20–27), EPT richness (0.5–5), and Shannon diversity (1.61–2.29) in bucket samples, with Sub1 consistently reflecting the most degraded conditions, and Catch having the highest diversity and richness of sensitive EPT taxa. Although net samples captured more individuals, richness and diversity metrics were slightly higher in the quantitative, bucket samples. Density of macroinvertebrates within bucket samples ranged from 94 to 48,648 individuals m^−2^ (average density 2,892 individuals m^−2^) and biomass ranged from 5.5 to 81,951 mg AFDM m^−2^ (average biomass 1,986 mg AFDM m^−2^) across samples.

**Figure 5:**
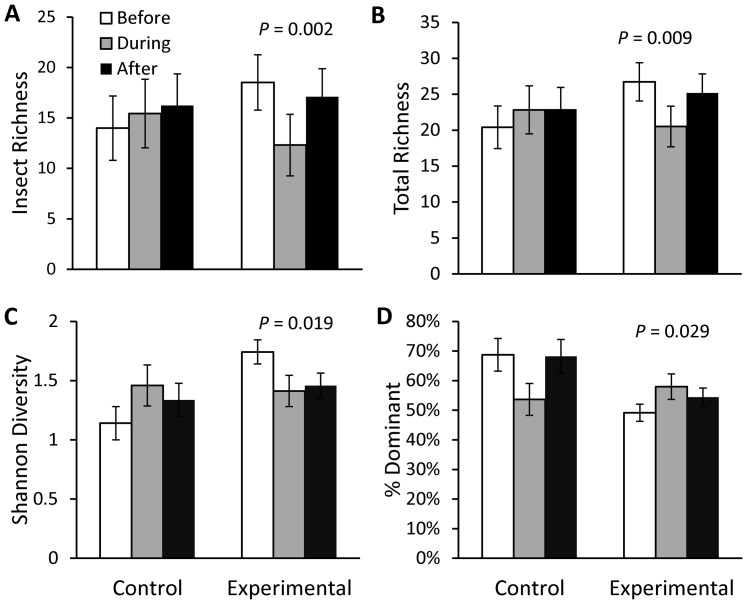
Macroinvertebrate variables before, during, and after treatment for control and experimental sites. Mean (± SE) back-transformed values are reported for insect richness (A) and total richness (B) based on riffle bucket samples, and Shannon diversity (C) and % dominant (D) based on biomass values for riffle bucket samples. *P*-values reflect results of ANOVA for Group*Period interaction; only significant biotic models (*P*<0.05) are included (Table 4).

There were few significant effects of treatment (Group*Period) on the individual macroinvertebrate abundance and biomass variables (Table 4). Insect richness (Fig. 5A) and total richness (Fig. 5B) from bucket samples tended to increase through time in the Control sites, whereas the Experimental sites had lower richness in the During period compared to Before and After. Shannon diversity (based on biomass from bucket samples) was highest in the During period for the Control sites and Before treatment for the Experimental sites (Fig. 5C). Percent dominant taxon based on biomass from bucket samples (a variable that should increase with disturbance) was lowest in the During period for the Control sites and highest During treatment for the Experimental sites (Fig. 5D).

**Figure 6:**
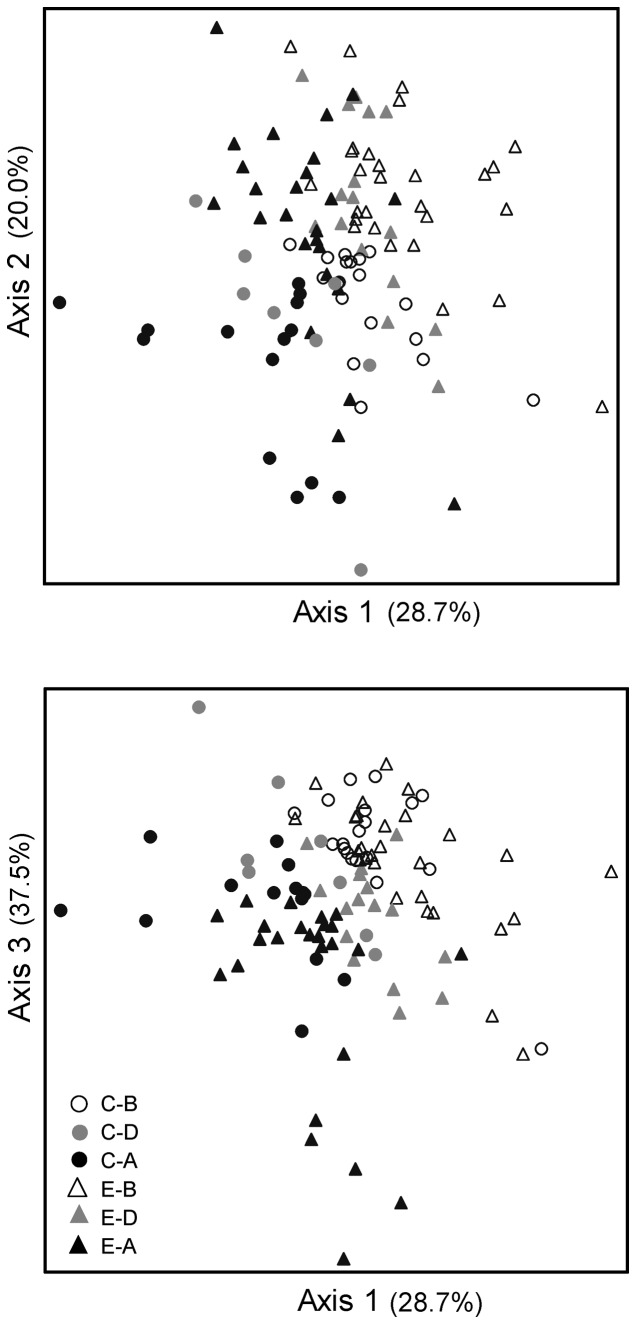
Non-metric multidimensional scaling ordination for macroinvertebrate abundances from bucket samples. Assemblages were different for Control (C) and Experimental (E) sites (Axes 2 and 3) and comparing Before (B), During (D), and After (A) installation of stormwater management (all axes combined; Table 5). The 3D solution explained 86.2% of the variation, and the final stress was 16.1.

**Figure 7:**
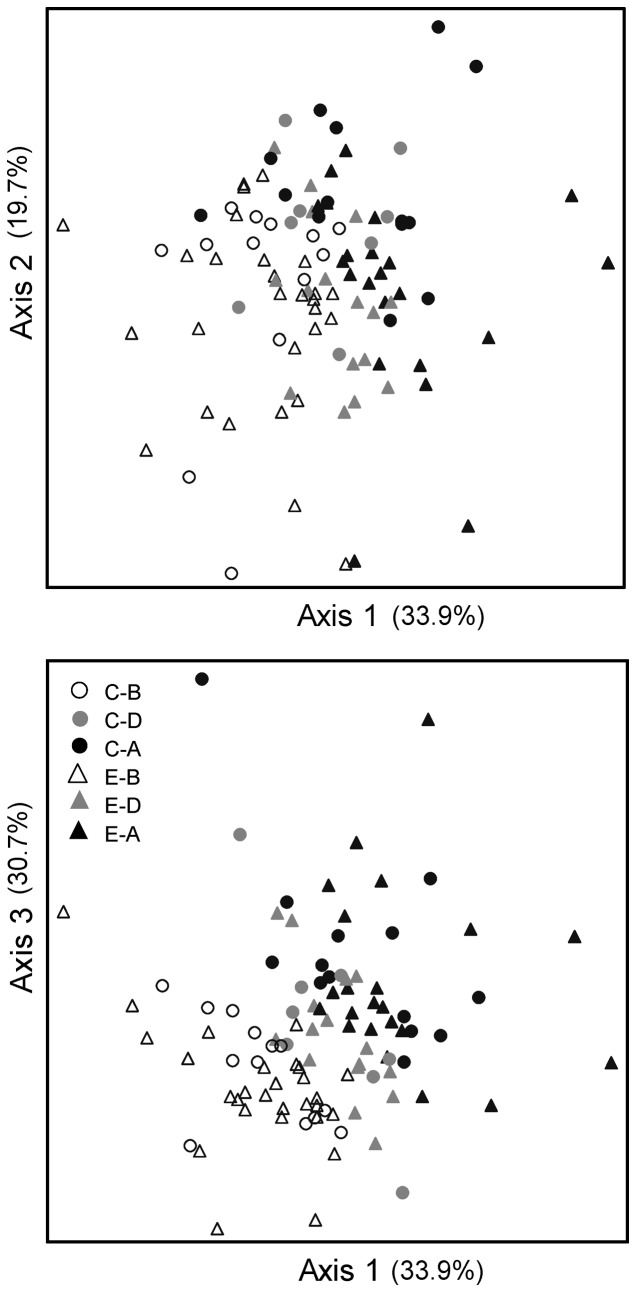
Non-metric multidimensional scaling ordination for macroinvertebrate biomass from bucket samples. Assemblages were different for Control (C) vs. Experimental (E) sites (Axis 2) and comparing Before (B), During (D), and After installation of stormwater management (Axes 1, 3, and all; Table 5). The 3D solution explained 84.3% of the variation, and the final stress was 15.8.

Macroinvertebrate assemblages collected from riffle habitats were distinct based on Group (Control vs. Experimental) and Site (Table 5) based on the ordination of taxa abundance (Fig. 6) and biomass (Fig. 7). There was also a significant Period effect for abundance (combined axes) and biomass (axis 1 and combined axes; Table 5). Only macroinvertebrate abundance axis 3 revealed a significant effect of stormwater treatment (Group*Period, *P* = 0.002, Table 5).

## Discussion

### Stream responses to rain garden and barrel installations

As expected, the installation of rain barrels and rain gardens on 30% of the properties in the Experimental catchments resulted in very few responses in stream water quality, periphyton, and macroinvertebrate metrics relative to Control sites. The few significant results that were detected should be interpreted with caution, given the high number of comparisons and potential risk of Type I error. Despite the high number of samples, with only four experimental and two Control sites there was low statistical power, a challenge of BACI designs [35]. Nonetheless, the few detected responses are notable given the study design and relatively small amount of stormwater runoff mitigated in the catchment.

There was a statistically significant effect of stormwater treatment on a few baseflow water quality variables, generally reflecting reduced water quality through time at Control sites. The small reduction in runoff volume from Before to After treatment [31] may have stabilized the water quality in the Experimental sites over a period of time when conditions in the Control sites were deteriorating. Rain gardens and barrels captured runoff, thereby reducing the likelihood of pollutant mobilization and transport, and potentially decreasing the total mass of pollutants delivered to streams during higher-flow storm conditions [49], [50]. In addition to capturing and detaining stormwater, rain gardens can play a role in filtration of pollutants [51], [52], and may contribute to overall reduced pollutant loading to streams, with some potential for improved baseflow water quality [49], although this mechanism was untested in our study.

There was no significant effect of treatment on the algal community, as examined through individual periphyton metrics and ordination of cell densities by taxon. This is not surprising given the lack of a response of nutrients (nitrogen and phosphorus) to treatment, and that these systems are not significantly nutrient limited [53]. The qualitative filamentous algal score was much higher in the Control sites in the During period (June 2007 through September 2008), which corresponded to the removal of trees and shrubs within the riparian zones at both Control sites. Loss of riparian cover increased the duration and intensity of light reaching the stream, potentially also increasing stream temperature locally, and could have triggered the increase in the relative proportion of filamentous green algae in the Control sites [53], [54]. The lower scores for the After period may be a reflection of algal sloughing during the After period, which had higher precipitation and flows compared to the During period.

The few significant treatment effects on the macroinvertebrate assemblage were not intuitive, and may be explained by multiple factors independent of the stormwater management. In the Control sites, there was an increase in richness and diversity through time, and lower percentage of dominant taxa in the During period. These patterns may reflect the periphyton responses, especially if increases in filamentous algae provided habitat, food, or increased nutrient uptake to support new taxa and higher diversity in the Control sites. In contrast, the Experimental sites demonstrated lower richness and diversity, and higher percentage of dominant taxa in the During period, compared to Before and After. It is possible that the reductions can be attributed to differences in low flow hydrology over the course of the study. Whereas high storm flows can directly alter macroinvertebrate communities through physical washout [55], small streams such as those in our study are more likely structured by seasonal variation in stream flow. The two Control streams (Sub4 and Sub5) had two of the three smallest catchment areas, and dried to pools nearly every summer, whereas the other sites remained perennial, which may explain why the Control sites had the lowest overall richness and diversity. However, in 2007 and 2008 (when the stormwater management devices were being installed), all six streams dried to pools in the summer. The lack of permanent flow and associated fluctuations in temperature likely resulted in loss of taxa in the Experimental sites that require flowing water, and these taxa may have already been missing from the Control sites [56], [57].

### Why were there so few responses to stormwater management?

Although the installation of rain gardens and rain barrels represented a widespread retrofit management effort, it is likely the number and capacity of installations were simply insufficient to elicit any response from the water quality or biotic measures. The management effort targeted runoff from rooftops and driveways on private properties, which comprised a majority (53.2%) of the total impervious area in the Shepherd Creek catchment. However, even if the 30% of properties that received treatments captured all of the runoff from rooftops and driveways on those properties (which we know was not the case), it would not reduce the effective impervious area (EIA) or DCIA in the subcatchments to below the threshold (1–14% EIA [6], 2% TIA [32]) of expected biotic impairment (See Fig. 2.2.2 in [58]). The range in impervious (11.2–19.9%) and forest (43.8–68.0%) cover across sites may have also masked detection of responses to treatments. Furthermore, our management approach did not address runoff from streets, which comprised 22.7% of the total impervious area, but had a proportionally higher amount of impervious cover directly connected to storm sewers [38]. Streets were therefore likely to have a disproportional impact on streams, and their lack of treatment may have masked the benefits provided by the rain gardens and rain barrels. Walsh et al. [59] demonstrated that it is possible for streams with ∼10% imperviousness to have good ecological condition if stormwater is infiltrated throughout the catchment. Thus, it is conceivable to achieve in-stream improvements in the Shepherd Creek catchment if we (1) increase the number of properties with management practices, (2) ensure all impervious surfaces on the property are routed to rain gardens, (3) increase the capacity of management devices, and (4) mitigate runoff from streets with high proportions of connected impervious cover.

In addition to the lack of hydrologic capacity of installed stormwater treatment devices, there are other possible reasons for the lack of water quality and biotic responses. First, there could have been an overwhelming influence of other stressors, despite the reduced stormwater volume. Although in-stream hydrology is tightly linked to water quality and biotic health in suburban and urban streams, water quality and other stressors (e.g., dispersal barriers, riparian forest loss, channelization) can shape biotic communities independently of stormwater runoff [9]. In the Shepherd Creek catchment, some of the properties have private septic tanks, and poorly maintained or malfunctioning systems can increase nutrients and bacteria, especially during low-flow conditions [60]. Road salt inputs were extremely high in the catchment, and although this can be partially mitigated by capturing runoff, salt concentrations and conductivity remained high despite restoration efforts. It is possible that these and other aquatic and terrestrial stressors were not mitigated by restoration efforts, thus preventing improvement in periphyton and macroinvertebrate assemblage integrity.

Over the seven-year study, there were many changes in the catchment unrelated to the project that may have masked responses associated with stormwater management. The Shepherd Creek project involved county and city organizations (e.g., Hamilton County Soil and Water Conservation District, Hamilton County Engineers Office, Cincinnati Metropolitan Sewer District, and Cincinnati Parks) in effort to maximize potential effectiveness of the project. Despite this, a few road maintenance, sewer maintenance, and tree removal projects occurred during the study period. These changes can increase variability in response variables and reduce the potential to detect improvements in the catchment. Because tree removal (to improve road visibility in Sub4 and for management of invasive species in Sub5) occurred at the same time as installation of stormwater management devices, it was difficult to separate the causes of any biotic responses. In addition, landowners likely made changes in their landscaping, watering, and other practices independently of the project. Given that it is unrealistic to prevent these non-target changes in suburban catchments, future studies will likely have to do additional improvements, matching the scope of management to the type and extent of disturbance, in order to detect a response.

The lack of detectable responses may also be explained by the high spatial and temporal variability of the biotic variables, which is typical in small, hydrologically-complex urban catchments [61]. In the spatial dimension, there are large differences in macroinvertebrate [46] and periphyton [62] taxa found across habitats. By targeting sample collection to riffle habitats (macroinvertebrate bucket samples) and hard substrates (periphyton samples), our study minimized this within-reach variation, although habitat was still likely an important factor influencing differences across sites. We observed high intra- and inter-annual variability in both the periphyton and macroinvertebrate assemblages. Although these were accounted for in the statistical model (Round), the additional variable in the model can minimize the power to detect a response, a disadvantage of this hybrid designed-observational study with numerous unavoidable nuisance effects. Moreover, research suggests that weather variability (on a small scale) and climate variability (on a larger scale) may drive assemblages [56], [57], [63], [64] and override responses to localized hydrologic management in some years. As mentioned before, we experienced drought conditions during the installation phase (late 2007 & early 2008), which may explain biotic responses in the During period. It is likely the low flows, combined with other stressors (water quality, temperature, sedimentation) in the catchment, interacted in complex ways to control biotic assemblages and ultimately prevent any detectable response to stormwater management.

Whereas improvements in hydrology were expected almost immediately following restoration, subsequent improvements in water quality and biotic integrity may take much longer. This study included three years of post-restoration monitoring after the initial installations (Phase 1), and only two years of monitoring after all of the installations were complete (i.e., following Phase 2). Existing, sediment-bound pollutants may take several years to process before streams experience improved water quality from reduced loading [65] and contaminated groundwater reservoirs can maintain high levels of contaminates in streams decades after the pollutant source has been eliminated [66]. Although periphyton have relatively short life cycles and are more sensitive to short-term shifts in water quality than macroinvertebrates [39], [62], their assemblages are structured by habitat and substrate, neither of which changed during our study. Similarly, macroinvertebrates may display a delayed response to increased detention of stormwater runoff because it takes time for critical resources (e.g., food, habitat) to improve [67]. Furthermore, the multi-year life cycles of many macroinvertebrates and their modes of dispersal suggests that recovery may take several years [39]. Even in studies where in-stream habitat enhancement has restored habitat diversity, some researchers have shown limited recovery of macroinvertebrates that can be explained by other, persistent stressors [67]–[69]. Even if instream conditions are suitable, aquatic invertebrates that have terrestrial adults need good riparian habitat and dispersal corridors across the landscape to persist [70].

### Conclusions

Although this study represents a sizeable effort to control stormwater runoff on private properties throughout a catchment, the stream responses to the retrofit management were limited to localized responses of a few variables. These results are not surprising given the number of rain gardens and rain barrels and the capacity of these stormwater devices relative to the impervious surfaces in the catchment. There is an obvious need for additional controlled studies where stormwater management practices are installed at higher densities to capture a greater volume of stormwater to determine the extent of stormwater management necessary to improve ecosystem health. A large-scale stormwater restoration is currently underway in the Little Stringybark Creek catchment in Melbourne, Australia [27], and more studies of this scope are needed despite the logistical and financial challenges of implementing and monitoring catchment-scale restoration [26].

The focus of this paper was on the effects of stormwater management on stream water quality and biota. However, the success of stream restoration should not only be measured in terms of improved ecosystem health [71], but in other benefits that can be derived. This study took a multidisciplinary approach to designing and implementing catchment-scale management, providing economic and social benefits that extend beyond the ecosystem responses [28]. The auction revealed substantial landowner interest and the potential for mitigating stormwater at much lower costs than centralized options. There were additional contributions to ecosystem services such as flood protection and water supply that extend beyond stream ecosystem benefits. For example, the 165 rain barrels installed resulted in water savings in cases where residents used the outside water source for watering that would otherwise come from potable sources. The 81 rain gardens included only native plant taxa, so the installations contributed to increases in native flora and wildlife habitat in the neighborhood. Additional benefits included generating public awareness of stormwater issues and the connection between human activities and environmental quality. Overall, it is clear that management efforts designed to mimic natural ecosystems will provide a variety ecosystem and other benefits, yet the extent of retrofit stormwater management necessary to restore healthy streams remains to be determined.
